# Supplementary material for: The Risk of SARS-CoV-2 Infection in Pregnant Women: An Observational Cohort Study Using the BIFAP Database
Source: Healthcare (Basel). 2022 Dec 2;10(12):2429. doi: 10.3390/healthcare10122429 (PMC9777798; doi:10.3390/healthcare10122429)
Supplement: Supplementary file 1 [file healthcare-10-02429-s001.zip › healthcare-2042996-supplementary.pdf]

Supplemental Table S1. Baseline characteristics of both cohorts and clinical course of SARS-COV2 among groups

Characteristics	Comparison Cohort N= 41,2740		Pregnancy Cohort N=10,3185	
**Hospitalized COVID**	**704**	2.8	549	6.4
1st trimester	na	na	12	2.9
2nd trimester	na	na	5	1.2
3rd trimester	na	na	394	95.9
**ICU COVID**	19	0.1	5	0.1
**Symptoms recorded in the database (+/- 7 days)**				
Cough	74	0.3	26	0.3
Anosmia	11	0.0	9	0.1
Thoracic pain	9	0.0	1	0.0
Abdominal pain	19	0.1	13	0.2
Headache	72	0.3	27	0.3
Diarrhea	53	0.2	14	0.2
Muscle or body aches	806	3.2	334	3.9
Fever	282	1.1	85	1.0
Nausea	22	0.1	11	0.1
Nasal Congestion	13	0.1	9	0.1
**Complications (0-28 days)**				
Bronchitis	11	0.0	2	0.0
Pneumonia	49	0.2	19	0.2
Thrombosis	6	0.0	4	0.0
Valvopathy	1	0.0	0.0	0.0

There were no recorded patients having tiredness, Bronchiolitis, Distress respiratory syndrome, Disseminated intravascular coagulation and myocarditis.
